# Interdisciplinarity and Infectious Diseases: An Ebola Case Study

**DOI:** 10.1371/journal.ppat.1004992

**Published:** 2015-08-06

**Authors:** Vanessa O. Ezenwa, Anne-Helene Prieur-Richard, Benjamin Roche, Xavier Bailly, Pierre Becquart, Gabriel E. García-Peña, Parviez R. Hosseini, Felicia Keesing, Annapaola Rizzoli, Gerardo Suzán, Marco Vignuzzi, Marion Vittecoq, James N. Mills, Jean-François Guégan

**Affiliations:** 1 Odum School of Ecology and Department of Infectious Diseases, College of Veterinary Medicine, University of Georgia, Athens, Georgia, United States of America; 2 DIVERSITAS, Muséum National d'Histoire Naturelle, Paris, France; 3 UMI IRD/UPMC 209 UMMISCO, Bondy, France; 4 INRA, UR346 Épidémiologie Animale, Saint Genès Champanelle, France; 5 UMR 5290 IRD-CNRS-Université de Montpellier, Centre IRD de Montpellier, Montpellier, France; 6 CESAB—Centre de Synthèse et d’Analyse sur la Biodiversité, Aix-en-Provence, France; 7 EcoHealth Alliance, New York, New York, United States of America; 8 Biology Program, Bard College, Annandale-on-Hudson, New York, United States of America; 9 Fondazione Edmund Mach, Department of Biodiveristy and Molecular Ecology, San Michele all’Adige (TN), Italy; 10 Facultad de Medicina Veterinaria Zootecnia, Universidad Nacional Autónoma de México, Ciudad Universitaria, México, Distrito Federal, México; 11 Institut Pasteur, Viral Populations and Pathogenesis, CNRS UMR 3569, Paris, France; 12 Centre de recherche de la Tour du Valat, Le Sambuc, Arles, France; 13 Population Biology, Ecology, and Evolution Program, Emory University, Atlanta, Georgia, United States of America; The Fox Chase Cancer Center, UNITED STATES

High-profile epidemics such as Ebola, avian influenza, and severe acute respiratory syndrome (SARS) repeatedly thrust infectious diseases into the limelight. Because the emergence of diseases involves so many factors, the need for interdisciplinary approaches to studying emerging infections, particularly those originating from animals (i.e., zoonoses), is frequently discussed [1–4]. However, effective integration across disciplines is challenging in practice. Ecological ideas, for example, are rarely considered in biomedical research, while insights from biomedicine are often neglected in ecological studies of infectious diseases. One practical reason for this is that researchers in these fields focus on vastly different scales of biological organization (Fig 1), which are difficult to bridge both intellectually and methodologically. Nevertheless, integration across biological scales is increasingly needed for solving the complex problems zoonotic diseases pose to human and animal well-being. Motivated by current events, we use Ebola virus as a case study to highlight fundamental questions about zoonoses that can be addressed by integrating insights and approaches across scales.

**Fig 1:**
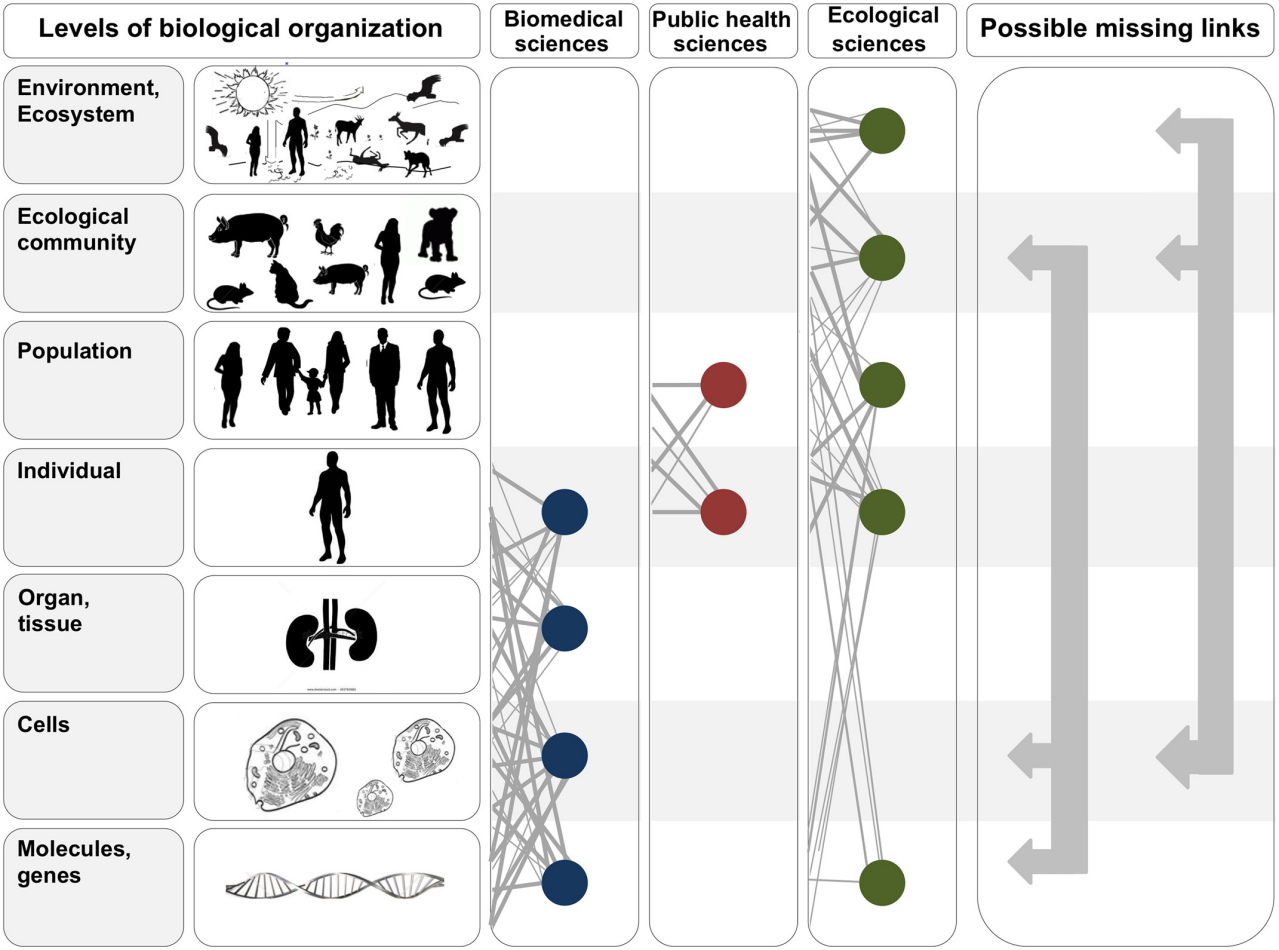
In the two leftmost panels, we depict the hierarchy of biological organization, from molecules and genes to ecosystems. Each level of the hierarchy reflects an increase in organizational complexity, with each level being primarily composed of the previous level’s basic units. Middle panels illustrate how the study of interactions between infectious disease agents and their hosts differs across the biomedical, public health, and ecological sciences. Specifically, biomedical sciences typically focus on lower- and medium-scale levels of biological organization (e.g., molecules, genes, and organs). In contrast, public health and ecological sciences typically focus on medium- and higher-scale levels of organization (individual, population, community, ecosystem, and environment). The filled circles and solid lines connecting the circles illustrate key cross scale biological interactions studied within each field. The right panel shows example knowledge gaps that can emerge from the “typical” segregation of research activities across the three fields. To better integrate our understanding of the causes and consequences of zoonotic infectious diseases, researchers must begin focusing on these types of missing links.

## Ebola Severity: A Cell-to-Ecological Community Perspective

Zaire ebolavirus (EBOV), the virus responsible for the 2014 Ebola outbreak in West Africa, causes a deadly haemorrhagic disease in humans with case fatality rates ranging from 60%–88% [5]. Although well-known for its lethality, Ebola severity is variable at the individual level; some people die of infection, some survive, and some never develop symptoms [6–8]. Asymptomatic infection is poorly understood but may have important implications for how EBOV spreads. After a 1996 outbreak in Gabon, one study found that 45% of household contacts of symptomatic patients never developed disease symptoms despite becoming infected with the virus and mounting EBOV-specific immune responses [7]. Intriguingly, asymptomatic infection might also result from contact between humans and animals. As an example, a 2010 serological survey of over 4,000 people from 220 villages in Gabon found that 15% of people overall, and 19% of those in forested regions, had EBOV-specific immunoglobulin G (IgG) antibodies [9]. Detection of EBOV-specific T cell responses in a subset of IgG+ individuals corroborated that these individuals were exposed to EBOV. Based on the known epidemiology of Ebola in Gabon, the authors ruled out human-to-human transmission as a sufficient explanation for the high antibody prevalence. Instead, they hypothesized that human–animal contact, specifically human contact with noninfectious virus particles in the environment (e.g., by eating or handling fruit contaminated with the saliva of infected bats), may have triggered virus-specific immune responses. If the immune responses detected in Gabon are protective against subsequent EBOV infection, large-scale phenomena occurring at the level of the ecological community might interact with molecular and cellular-level processes to influence the severity of any given Ebola outbreak.

Using an epidemiological model, Bellan et al. [10] showed that accounting for asymptomatic infections that induce protective immunity reduced Ebola incidence projections for Liberia by 50%. Ultimately, the relative frequency of protective asymptomatic infections determines the size of this effect. Although the model was predicated on asymptomatic infection occurring during human-to-human transmission, asymptomatic cases that arise from environmental exposure, as hypothesized by Becquart et al. [9], could have similar dampening effects on epidemic spread. The frequency of such environmental exposure would depend on the animal community in a region. If certain bat species are the natural reservoirs of EBOV [11], their presence, relative abundance, and behaviour could all affect the frequency with which humans come into contact with them and thereby develop “environmentally-induced” immune protection. Of course, human contact with bats also triggers Ebola outbreaks [12], so understanding the context in which human–bat contact is protective (e.g., induces asymptomatic infection and immunity) rather than hazardous (e.g., causes symptomatic infection and epidemic spread) requires investigating phenomena occurring in both humans and bats, from the drivers and frequency of contact between humans, bats, and other relevant species to the characteristics of host cell–virus interactions upon contact.

## Ecosystem Dynamics, Viral Evolution, and Human Epidemics

The Ebola outbreak in West Africa and punctuated outbreaks in Central Africa since the 1970s raise fundamental questions about what drives disease spillover to humans. Ebola outbreaks are not limited to human populations. Wildlife die-offs occur routinely before or during human epidemics, indicating that the virus circulates in a range of other mammal species, including great apes and forest antelopes [13–16]. Even though these species are not considered natural reservoirs, circulation of EBOV in these animals still has implications for human disease. First, human contact with these species can directly trigger disease outbreaks [17]. Second, these animals might affect spillover risk by influencing rates of virus evolution. Phylogenetic analysis of EBOV in great apes [18] suggests that genetic variation can accumulate rapidly during EBOV transmission in these populations. Importantly, virus evolution in animal hosts may facilitate the emergence of strains that spread more efficiently to humans or that cause more severe disease.

Although unknown for EBOV, the idea that virus circulation in wild species can drive changes that impact human–virus interactions has support for other RNA viruses such as SARS coronavirus and influenza A virus (see Table 1) [19,20]. Given evidence from these other viruses, understanding if and how animal hosts affect EBOV evolution is crucial. Doing this requires studies that connect large-scale environmental and ecosystem processes to small-scale genetic and molecular processes. For example, food web or habitat structure may determine the diets of target wildlife species, and host nutrition could affect rates of infection, virus replication, and shedding. Likewise, contact rates among species determine levels of cross species virus transmission, which may influence virus mutation or recombination rates. These examples, though speculative, highlight how cross scale chains of events might influence disease emergence in humans.

**Table 1:** Examples of zoonotic disease systems in which cross scale research has contributed to key insights about infection dynamics.

Disease/Pathogen	Question(s)	Scales of Research	Key insights	References
Influenza / influenza A virus (global, 1800s–present)	What drives the emergence of pandemic strains?	Environment	In temperate regions, absolute humidity interacts with levels of susceptibility and human-to-human contact patterns to influence the timing of pandemic influenza outbreaks.	[20–24]
		Population	The diversity of influenza virus strains circulating in bird populations is driven by both population mechanisms (transmission ecology) and pathogen characteristics (substitution rates).	
		Gene	Pandemic virus strains in human populations have arisen from the introduction of genes from avian and swine influenza viruses.	
		Molecule	Under experimental conditions, molecular changes in the hemagglutinin (HA) protein from highly pathogenic avian influenza (H5) can facilitate efficient mammal-to-mammal transmission.	
SARS / SARS coronavirus (SARS-CoV) (global pandemic, 2002–2003)	What is the transmission cycle that caused the global SARS epidemic?	Community	Isolation and phylogenetic analysis of virus from multiple bat species identified bats as the natural reservoir for SARS.	[25–31]
		Population	Specific individuals with disproportionately high contact rates (super spreaders) were responsible for a majority of virus transmission events in humans.	
		Cell	Single amino acid substitutions in SARS-CoV of palm civet origin can enhance viral entry into human angiotensin-converting enzyme 2 (ACE2) receptor-expressing cells.	
		Gene	The receptor binding domain of the SARS-CoV Spike protein underwent rapid evolution in nonreservoir “intermediate” hosts such as palm civets, potentially facilitating virus transmission to humans.	
Hendra virus, (Australia, 1994–present)	What factors influence disease spillover from bat reservoirs to horses and humans?	Environment/ecosystem	Shifting bat distributions and changes in migratory behaviour are facilitated by anthropogenic habitat modification.	Reviewed in [32]
		Population/individual	Temporal and spatial pulses of virus shedding in bats may be influenced by individual host traits such as nutritional stress or reproductive status. The amount of virus released in any area is a function of local bat density and the shedding status of individual bats in the population.	
		Cell	Only a subset of exposed horses are identified as spillover cases. Upon exposure, some horses eliminate infection with a strong innate immune response and some mount an acquired response after asymptomatic infection or clinical disease, while others experience fulminating infection.	
Hantavirus pulmonary syndrome / Sin Nombre virus (SNV) (southwestern United States, 1993)	Was this a new disease agent? What caused spillover to humans?	Environment/ecosystem	Increased precipitation due to El Niño promoted enhanced primary production in the spring.	Reviewed in [33–34]
		Population	Rodent reservoir populations greatly increased in size as a result of improved food availability.	
		Individual	Most human exposures occur in peridomestic environments where the host of SNV, deer mice, thrive. The breeding season of deer mice is up to two months longer in peridomestic settings, and infection rates tend to be higher in these environments.	
		Gene	Phylogenetic reconstruction of virus samples collected from cryogenically preserved mice determined that the virus was present in rodents prior to the 1993 human outbreak.	
Lyme disease / *Borrelia burgdorferi* (northeastern US, 1975–present)	What accounts for temporal and spatial variation in human infection risk?	Community	Oak tree masting influences acorn abundance, which determines the future population density of reservoir hosts. The diversity of hosts available on which ticks can feed determines both the abundance of ticks and the infection rates of these ticks.	[35–39]
		Individual	White-footed mice, which are the hosts most likely to pass the Lyme bacterium to feeding ticks, do not show negative effects of infection, suggesting that the bacterium might be a mutualist rather than a parasite on this key host species.	
		Cell	Mice show a weak antibody response to experimental infection, but a strong innate response, suggesting that they might trade investment in long-term adaptive immunity for investment in short-term immunity.	

## Towards a More Integrative Future

The Ebola outbreak in West Africa reminds us that zoonotic diseases continue to be a major threat. The benefits of cross scale research are evident for several high-profile zoonoses (Table 1). Nevertheless, this type of work is far from the norm, and successful integration of research approaches across vastly different biological scales remains challenging. A first step toward greater integration involves student training. Training programs in infectious disease typically focus on a single or narrow range of biological scales, but more crosscutting approaches are needed. Training grants focused on multiscale literacy in infectious disease research should be a priority for funding agencies, for example. Professional societies could also lead the way by sponsoring workshops, symposia, and other events on integration across disciplines. The involvement of professional societies has the added benefit of allowing infectious disease researchers to expand their perspectives beyond their years of formal training. Updating our collective mind-set in these and other ways will put us in a much better position to tackle the next zoonotic disease threat.
